# Melatonin Protects Intact Rat Ovarian Transplantation via the MT1/Nrf2/ARE Pathway

**DOI:** 10.3390/cells14201588

**Published:** 2025-10-13

**Authors:** Lingyun Xie, Shanshan Wang, Yuling Wu, Xuyin Zhang, Yan Ding

**Affiliations:** Obstetrics & Gynecology Hospital of Fudan University, Shanghai Key Lab of Reproduction and Development, Shanghai Key Lab of Female Reproductive Endocrine Related Diseases, Shanghai 200433, China; xielingyun0602@163.com (L.X.); wangshanshan10214@fckyy.org.cn (S.W.); wuyuling10295@fckyy.org.cn (Y.W.); zhangxuyin1564@fckyy.org.cn (X.Z.)

**Keywords:** melatonin, in vivo study, ovarian transplantation, rat models, oxidative stress, ischemia-reperfusion injury, anticancer therapy-related infertility, fertility preservation, anti-inflammatory

## Abstract

Cryopreservation and transplantation of intact ovaries offer a promising approach to fertility restoration in cancer patients. However, ischemia–reperfusion injury following transplantation significantly impairs graft function. This study aimed to evaluate the protective effects of melatonin and elucidate its underlying mechanisms of action, including antioxidant and anti-inflammatory properties. Intact ovaries from 8 to 12-week-old LEWIS rats were cryopreserved and subsequently transplanted. Melatonin (25 mg/kg and 50 mg/kg) was administered daily from day 1 to day 4 postoperatively. Estrous cycle recovery and ovarian histology were examined, along with measurements of hormone concentrations, antioxidant activity, and inflammatory mediators. The oxidative stress response, particularly the nuclear factor erythroid 2-related factor 2 (Nrf2)/antioxidant response elements (ARE) signaling pathway—including Nrf2, Kelch-like ECH-associated protein 1 (Keap1), and sMafg—was investigated to elucidate melatonin’s protective mechanisms. The roles of melatonin receptors and Nrf2 were investigated using specific receptor antagonists (Luzindole, 4P-PDOT) and an inhibitor (ML385) to confirm the involvement of the MT1/Nrf2/ARE pathway. As a result, rats treated with high-dose melatonin (50 mg/kg) exhibited accelerated estrous cycle recovery, reduced follicular loss, improved serum hormone levels, enhanced antioxidant capacity in serum and ovarian tissue, and decreased levels of inflammatory cytokines. Furthermore, melatonin exerted its antioxidant and anti-inflammatory effects through activation of the Nrf2/ARE signaling pathway via the MT1 receptor. These protective effects were abolished by the inhibition of either Nrf2 or MT1 receptor. In conclusion, these findings demonstrate that melatonin mitigates oxidative stress and inflammatory damage in intact transplanted ovaries through the MT1/Nrf2/ARE signaling axis, thereby preserving ovarian function post-transplantation.

## 1. Introduction

Cancer remains the second leading cause of mortality among women globally [1,2,3]. Advances in oncologic therapies have substantially improved survival rates in female patients; however, these treatments, including radiotherapy and chemotherapy, are often detrimental to reproductive function. Following cancer diagnosis and treatment, women are reported to have a 38% lower likelihood of achieving pregnancy [4]. Specifically, radiation therapy has been shown to induce premature menopause in a dose-dependent manner [5], while chemotherapeutic agents disrupt the dual endocrine and reproductive functions of the ovary [6,7]. A key mechanism underlying chemotherapy-induced ovarian dysfunction is the induction of DNA damage and apoptosis in ovarian follicles, ultimately depleting the follicular reserve [8].

To mitigate fertility loss, several preservation strategies have been developed, including oocyte and embryo cryopreservation, as well as ovarian tissue cryopreservation, which involves surgical extraction of ovarian tissue [9]. Despite these advances, significant follicular loss following transplantation—primarily due to ischemia–reperfusion injury (IRI) and follicular overactivation—remains a critical limitation [10,11]. Additionally, follicular loss has been linked to apoptosis, hypoxia, oxidative stress, and pyroptosis in oocytes and granulosa cells (GCs) [12,13]. Consequently, ongoing research aims to develop effective strategies to mitigate IRI-associated damage in transplanted ovarian tissue.

Endocrine and metabolic factors play important roles in post-transplant outcomes. For example, vitamin D sufficiency after transplantation is associated with improved graft and patient outcomes across multiple organ types [14,15,16,17], and vitamin D protects tissues from oxidative damage and supports healing processes [18,19]. Melatonin, secreted by the pineal gland, possesses well-established immunomodulatory and antioxidant properties [20,21]. It has demonstrated therapeutic potential in organ transplantation by attenuating uterine ischemia–reperfusion injury through its anti-inflammatory and antioxidative effects [22,23]. Although melatonin has been shown to improve the quality of ovarian grafts [24], the mechanisms underlying its influence on follicular viability, hormonal regulation, and long-term graft functionality remain poorly understood.

The primary objective of this study was to investigate the protective effects of melatonin on intact ovarian transplantation, with a particular focus on its anti-inflammatory and antioxidative mechanisms of action.

## 2. Materials and Methods

Our literature review and discussion were primarily based on studies published within the last 10 years to ensure the most current scientific context, with the exception of foundational/seminal works and established methodological papers that are critical for this field.

### 2.1. Animals

The surgery was performed according to the protocol described by Wang X et al. [25], which entailed the intact transplantation of a uterine horn, fallopian tube, and ovary onto the recipient aorta and vena cava. Following the transplantation, recipient rats underwent bilateral oophorectomy, and intact ovaries from 8 to 12-week-old donor rats were harvested and cryopreserved in liquid nitrogen. The intact rat ovaries were cryopreserved in liquid nitrogen for a minimum of 2 weeks before transplantation.

A total of 30 recipient rats were randomly assigned to six experimental groups: G1: Surgery + saline (control group); G2: Surgery + low-dose melatonin (25 mg/kg; MedChemExpress, Monmouth Junction, NJ, USA); G3: Surgery + high-dose melatonin (50 mg/kg); G4: Surgery + melatonin + nuclear factor erythroid 2-related factor 2 (Nrf2) inhibitor (ML385; Sigma, St Louis, MO, USA); G5: Surgery + melatonin + MT1 melatonin receptor (MT1) antagonist (Luzindole; Sigma, MO, USA); G6: Surgery + melatonin + MT2 melatonin receptor (MT2) antagonist (4P-PDOT; Sigma, MO, USA). In groups G2–G6, the respective treatments were administered intraperitoneally at 18:00 on the day preceding transplantation and continued through postoperative day 4. Rats were euthanized by CO_2_ inhalation on day 4 post-transplantation, and blood and ovarian tissue samples were collected for biochemical analysis. All procedures were approved by the institutional ethics committee (Approval No. 202209027Z). Animals were randomly allocated to experimental groups. All investigators involved in outcome assessment were blinded to the treatment allocations to minimize bias.

### 2.2. Endocrine Function

Daily vaginal exfoliative cytology was conducted beginning on the first day post-transplantation to monitor estrous cycle progression. Vaginal smears were obtained using saline-soaked swabs, fixed on slides, and stained with Giemsa solution. The slides were examined under a light microscope at 10× and 40× magnification. The stages of the estrous cycle were classified based on the predominant cell types observed: (1) proestrus—predominance of nucleated epithelial cells; (2) estrus—predominance of non-nucleated keratinized cells; (3) metestrus—equal proportion of leukocytes and keratinized epithelial cells; (4) diestrus—presence of all cell types in low numbers.

On day 4 post-transplantation, whole blood samples were collected, allowed to clot for 15 min, and centrifuged at 3000 rpm for 10 min to obtain serum. Hormone concentrations (progesterone, follicle-stimulating hormone (FSH), anti-Müllerian hormone (AMH), and estradiol) and inflammatory cytokines (tumor necrosis factor (TNF)-α and interleukin (IL)-6) were quantified using ELISA kits (Wuhan Huamei Biological Engineering Co., Ltd., Wuhan, China), following the manufacturer’s protocols. Absorbance was measured within 5 min using a microplate reader (Denley Dragon Wellscan MK3, Thermo, Vantaa, Finland) at 450 nm.

Fresh ovaries from the saline, low-dose melatonin, high-dose melatonin, and normal control groups were retrieved, fixed in 4% paraformaldehyde for 1 h, embedded in paraffin blocks, and serially sectioned at a thickness of 4–5 μm. Sections were stained with hematoxylin and eosin (H&E), mounted, and examined under a light microscope (Olympus-BX53 upright fluorescence microscope, Olympus Corporation, Tokyo, Japan) at 400× magnification to assess ovarian morphology and to quantify follicles at different developmental stages (Operating microscope: Thermo Finland Double Binocular Operating Microscope GX.SS.22-3, Shanghai Medical Optical Instrument Co., Ltd., Shanghai, China).

### 2.3. Measurement of Reactive Oxygen Species (ROS) in Ovarian Tissue

Fresh ovarian tissue samples (50 mg) from recipient rats were rinsed with PBS and cut into 5 mm × 5 mm pieces. Each sample was homogenized in 1 mL of Homogenization Buffer A using a tissue homogenizer and centrifuged at 100× *g* for 3 min at 4 °C. The supernatant was collected, and 200 μL was mixed with 2 μL of DHE probe in a 96-well plate. The mixture was incubated at 37 °C for 15–30 min in the dark. Fluorescence intensity was measured using a fluorescent enzyme marker at an excitation wavelength of 488–535 nm and an emission wavelength of 610 nm. Additionally, 50 μL of the supernatant was diluted approximately 30-fold with PBS, and 100 μL of the diluted sample was used for protein quantification. ROS levels in ovarian tissue were expressed as relative fluorescence units (RFU) per mg of protein.

### 2.4. Measurement of Serum ROS

Blood samples from recipient rats were allowed to stand at room temperature for 30 min, followed by centrifugation at 10,000 rpm for 5 min at 4 °C. The upper serum layer was collected and incubated with the O12 probe at 37 °C for 15 min in the dark. Fluorescence intensity was recorded at an excitation wavelength of 488 nm and an emission wavelength of 520 nm (Serum ROS Kit, Beyotime Biotechnology, Shanghai, China).

### 2.5. Measurement of Superoxide Dismutase (SOD), Glutathione (GSH), and Malondialdehyde (MDA) in Ovarian Tissue

Fresh ovarian tissues from recipient rats were cut into 5 mm × 5 mm pieces and homogenized using a tissue homogenizer. The homogenates were centrifuged at 10,000× *g* for 10 min at 4 °C, and the supernatants were collected. SOD, GSH, and MDA levels were measured using commercial assay kits in accordance with the manufacturer’s protocols.

### 2.6. Measurement of Total Antioxidant Capacity (TAC)

To assess total antioxidant capacity, blood samples were allowed to stand at room temperature for 30 min and centrifuged at 10,000 rpm for 5 min at 4 °C. The upper serum layer was collected and incubated with Cu^2+^ working solution at room temperature for 90 min on a shaker. Absorbance was measured at 570 nm using a microplate reader. TAC was determined based on a standard curve and expressed as antioxidant concentration (mmol) (Serum TAC Kit, Abcam, Cambridge, UK). Trolox (6-hydroxy-2,5,7,8-tetramethylchroman-2-carboxylic acid) in serial volumes (0, 12, 24, 36, 48, and 60 μL) was used as the standard to calculate the Trolox equivalent antioxidant capacity of the samples.

### 2.7. Measurement of Hydroxyl Radical Concentration in Ovarian Tissue Using the Fenton Assay

A suitable amount of fresh ovarian tissue was weighed and homogenized in 1 mL of extraction buffer on ice. The homogenate was centrifuged at 10,000× *g* for 10 min at 4 °C, and the supernatant was collected. The reaction mixture was prepared according to the manufacturer’s instructions, vortexed, and incubated at 37 °C for 1 h. The samples were then centrifuged at room temperature at 10,000 rpm for 10 min, and 200 μL of the supernatant was used for absorbance measurement at 536 nm. The absorbance values of the measurement group (At), control group (Ac), and blank group (A0) were recorded. The hydroxyl radical scavenging ratio was calculated as (At − Ac)/(A0 − Ac). Trolox and hemoglobin (Hb) at various concentrations were tested using the same method for comparison (Tissue Hydroxyl Radical Scavenging Kit, Solarbio, Beijing, China).

### 2.8. Nrf2 Protein Nuclear Translocation Assay in Ovarian Tissue

Paraffin-embedded tissue sections were rehydrated through a graded alcohol series and subjected to antigen retrieval in 10 mM sodium citrate buffer at 95 °C for 30 min. The sections were then permeabilized with 0.5% Triton X-100 and blocked with 5% bovine serum albumin (BSA) for 2 h. Slides were incubated with a primary antibody against Nrf2, followed by a fluorophore-conjugated secondary antibody. Nuclei were counterstained with DAPI for 10 min. After washing, the slides were mounted with anti-fade mounting medium and imaged using a fluorescence microscope (Carl Zeiss, Oberkochen, Germany).

### 2.9. Western Blot Analysis

Fresh ovarian tissue was finely minced and used to extract cytoplasmic and nuclear proteins using a commercial extraction kit (Cytoplasmic Cytosolic Nucleus Extraction Protein Kit, Beyotime Biotechnology, Shanghai, China). Protein concentrations were determined using the BCA Protein Assay Kit (Beyotime Biotechnology, Shanghai, China). Protein lysates were separated by SDS-PAGE on 12.5% gels (Yamei Bio, Shanghai, China) and transferred onto PVDF membranes. Membranes were blocked with 5% non-fat milk for 60 min at room temperature and incubated overnight at 4 °C with the following primary antibodies: rabbit anti-Nrf2, anti-SOD1, anti-heme oxygenase (HO)-1, anti-IL-6, anti-TNF-α (all from Cell Signaling Technology (CST), Danvers, MA, USA), anti-Histone H3 (CST, MA, USA), and anti-β-Actin (CST, MA, USA). Following incubation with HRP-conjugated goat anti-rabbit IgG (CST, MA, USA) for 2 h at room temperature, protein bands were visualized using an enhanced chemiluminescence (ECL) detection system. Densitometric analysis was performed using ImageJ software (Version 1.8.0), and the expression levels of target proteins were normalized to β-Actin or Histone H3 as appropriate.

### 2.10. Quantitative Reverse Transcription Polymerase Chain Reaction (qRT-PCR) Analysis

Total RNA was extracted from minced ovarian tissue using a commercial RNA purification kit (EZBioscience, Roseville, CA, USA), followed by reverse transcription using a color reverse transcription kit (EZBioscience, Roseville, CA, USA). qRT-PCR was conducted using SYBR Green Master Mix (EZBioscience, Roseville, CA, USA) on a real-time PCR system (Life Technologies, Carlsbad, CA, USA) under the following conditions: initial denaturation at 95 °C for 5 min, followed by 40 cycles of 95 °C for 10 s and 60 °C for 30 s. Gene expression levels were analyzed relative to internal controls. The primer sequences used were as follows:

Primer sequence (5′–3′)
Nrf2 F: GATCAGGCTCAGTCACTCGATAG
R: ACACTGTAACTCGGGAATGGAAA
HO-1 F: CAGGGTGACAGAAGAGGCTAAGA
R: TGGGATGAACTAGTGCTGATCTG
SOD1 F: GAGAGGCATGTTGGAGACCTG
R: TGTTTCTCGTGGACCACCATAG
IL-6 F: GGC CCT TGC TTT CTC TTC G
R: ATA ATA AAG TTT TGA TTA TGT
TNF-α F: CCAGGTTCTCTTCAAGGGACAA
R: GGTATGAAATGGCAAATCGGCT
KEAP1 F: GGACTTTCGTAGCCTCCATGAA
R: TAGCATTCCACACTGTCCAGAAA
sMafg F: CGTTGGGATCGGTCAGTTCA
R: CCACTTGCACTCTCGTCCAT
GAPDH F: CTGGAGAAACCTGCCAAGTATG
R: GGTGGAAGAATGGGAGTTGCT

### 2.11. Immunofluorescence Analysis

Ovarian tissue was fixed in 10% formalin for 24 h, embedded in paraffin, and sectioned at a thickness of 5 μm. The sections were dewaxed, rehydrated, and incubated overnight at 4 °C with primary antibodies against Nrf2 (1:400 dilution, CST, USA) and MLTR1A (1:400 dilution, Proteintech, Rosemont, IL, USA). Secondary antibodies conjugated to IgG (1:200 dilution) were subsequently applied. Nuclei were counterstained with 4,6-diamidino-2-phenylindole dihydrochloride (DAPI, Beyotime Biotechnology, Shanghai, China). Fluorescence imaging was performed using a Nikon fluorescence microscope (Nikon, Osaka, Japan).

### 2.12. Statistical Analysis

Statistical analyses were conducted using GraphPad Prism 9.4.1 (GraphPad Software, Boston, MA, USA, LLC). Data were presented as the mean ± standard deviation (SD) and were visualized using tables and bar graphs. One-way ANOVA followed by the LSD post hoc test was employed to evaluate differences among groups. A *p*-value of <0.05 was considered statistically significant.

## 3. Results

### 3.1. Melatonin Modulates Follicular Histology, Hormone Secretion, and the Estrous Cycle

**Figure 1:**
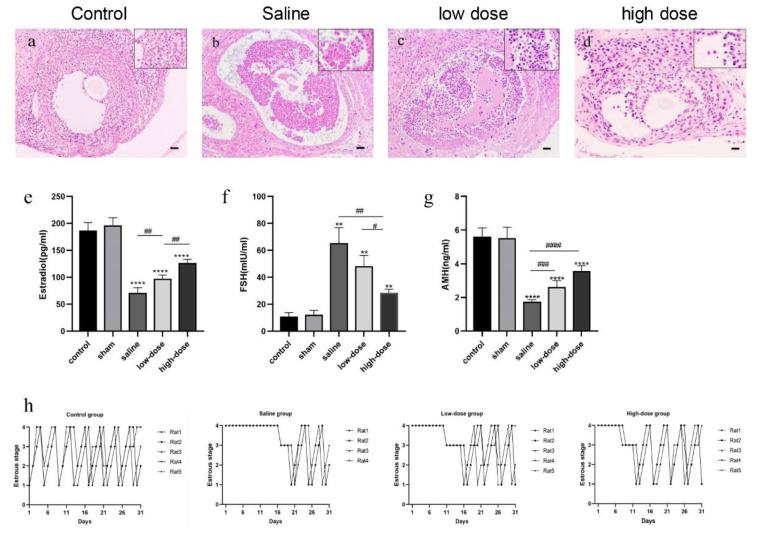
Effect of melatonin on the histology and number of follicles, hormone secretion, and estrous cycle. (**a**–**d**) Representative histological images of follicles on the fourth postoperative day of rats receiving intact ovarian transplantation. (**a**) Control group: fresh samples. (**b**) Saline group. (**c**) low-dose melatonin group. (**d**) high dose melatonin group; (**e**–**g**) Serum endocrine hormones on the fourth postoperative day of rats receiving intact ovarian transplantation (**e**) Serum estradiol, (**f**) serum FSH, (**g**) serum AMH levels in the control and sham-operated groups, (** *p* < 0.01, **** *p* < 0.001 vs. the sham-operated group; ^#^ *p* < 0.05, ^##^ *p* < 0.01, ^###^ *p* < 0.005, ^####^ *p* < 0.001 among the three transplantation groups). All experiments were performed in triplicate; (**h**) Recovery of estrous cycle of rats receiving intact ovarian transplantation in control, saline, low- and high-dose melatonin groups. Scale = 50 μm.

As illustrated in Figure 1, ovarian follicles in the saline group displayed abnormal morphology, including irregularly shaped oocytes and nuclear shrinkage (Figure 1b). Granulosa cells exhibited clustered atrophy and detachment from the basement membrane. In the low-dose melatonin group, follicular distortion was moderate, with irregular granulosa cell arrangement, basal membrane detachment, and vacuolar degeneration of perifollicular stromal cells (Figure 1c). Conversely, in the high-dose melatonin group (Figure 1d), follicular morphology closely resembled that of the fresh ovaries serving as non-transplanted controls (Figure 1a), exhibiting evenly distributed granulosa cells, round oocytes, and intact basement membranes.

These histological findings were supported by follicle counts, as shown in Table 1. The number of primordial, primary, secondary, and antral follicles was significantly reduced in the saline group compared to the fresh non-transplanted control. Melatonin administration preserved follicle numbers in a dose-dependent manner.

On the fourth postoperative day, the number of primordial, primary, and secondary follicles was significantly higher in the fresh non-transplanted control, low-dose, and high-dose melatonin groups compared to the saline group (* *p* < 0.05, ** *p* < 0.01). A higher number of antral follicles was found in the fresh non-transplanted control and high dose melatonin groups than in the saline group (** *p* < 0.01). Data were represented as means ± SD.

In terms of endocrine function, serum levels of estradiol (Figure 1e) and AMH (Figure 1g) were significantly reduced in all three transplantation groups compared to the fresh non-transplanted control (*p* < 0.001). However, both were significantly higher in the melatonin-treated groups relative to the saline group. Conversely, serum FSH levels (Figure 1f) were inversely correlated, with the fresh non-transplanted control group exhibiting the lowest levels and progressively higher levels observed in the high-dose, low-dose melatonin, and saline groups. Furthermore, the resumption of the estrous cycle was markedly delayed in the saline group (day 21 postoperatively). In contrast, the low- and high-dose melatonin groups exhibited earlier cycle resumption on postoperative days 16 and 11, respectively (Figure 1h).

### 3.2. Melatonin Regulates the Inflammatory Response in Intact Ovarian Transplantation

**Figure 2:**
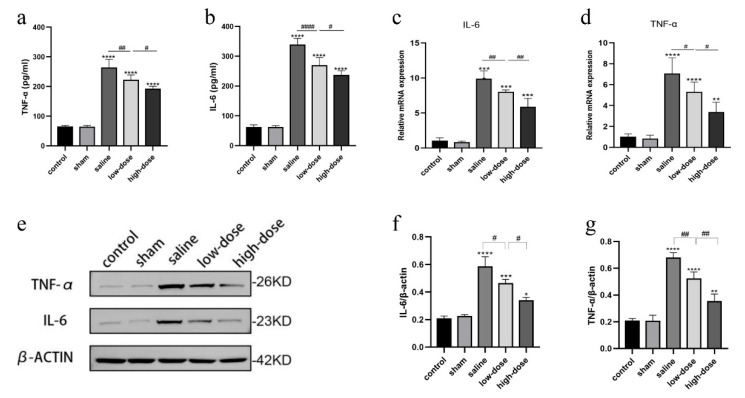
Effect of melatonin on inflammatory factors in the ovaries of rats receiving intact ovarian transplantation on the fourth postoperative day. (**a**,**b**) Serum IL-6 and TNF-α levels were three times higher in transplanted groups compared to the fresh non-transplanted control and sham-operated groups. (**c**,**d**) The mRNA levels of IL-6 and TNF-α in the ovarian tissues of the control group, sham, saline, low- and high-dose melatonin groups. (**e**) The expression of IL-6 and TNF-α in Western blot (**f**,**g**) Western blot quantization of IL-6 and TNF-α. (* *p* < 0.05, ** *p* < 0.01,*** *p* < 0.005, **** *p* < 0.001 vs. the control group; ^#^ *p* < 0.05, ^##^ *p* < 0.01, ^####^ *p* < 0.001 among the three transplantation groups). All experiments were performed in triplicate.

The effects of melatonin on the inflammatory response in cryopreserved and transplanted ovaries were evaluated. Elevated levels of serum inflammatory cytokines, including IL-6 and TNF-α, were observed in the saline and both melatonin treatment groups compared to the normal and sham-operated groups (Figure 2a,b). Notably, the high-dose melatonin group exhibited significantly lower levels of TNF-α and IL-6 than the saline and low-dose melatonin groups.

Further analysis using qRT-PCR and Western blotting revealed significantly increased mRNA and protein expression levels of IL-6 and TNF-α in all transplantation groups compared to the fresh non-transplanted control group (*p* < 0.01) (Figure 2c–g). However, these inflammatory markers were significantly reduced in the high-dose melatonin group compared to the saline and low-dose melatonin groups.

### 3.3. Melatonin Attenuates Oxidative Stress in Intact Ovarian Transplantation

**Figure 3:**
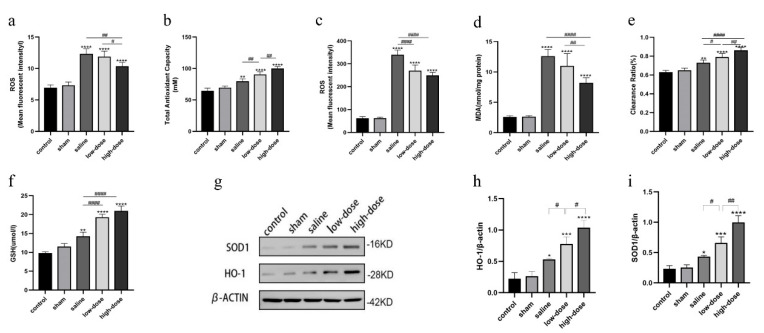
Melatonin effect on antioxidant function in rats receiving intact ovarian transplantation on the fourth postoperative day. (**a**) Serum ROS levels in the three transplanted groups, fresh non-transplanted control, and sham-operated groups, (**b**) Serum TAC levels in the three transplanted groups, fresh non-transplanted control, and sham-operated groups, (**c**–**f**) Tissue ROS, MDA, hydroxyl radical scavenging rate, and GSH levels in the three transplant groups, fresh non-transplanted control, and sham-operated groups. (**g**) Western blot results of the antioxidant enzymes SOD and HO-1 control, sham-operated, saline, and low- and high-melatonin groups (**h**,**i**). Western blot quantization results. (* *p* < 0.05, ** *p* < 0.01,*** *p* < 0.005, **** *p* < 0.001 vs. the sham-operated group; ^#^ *p* < 0.05, ^##^ *p* < 0.01, ^####^ *p* < 0.001 among the three groups of transplantation). All experiments were performed in triplicate.

The role of melatonin in modulating the oxidative–antioxidant balance was investigated by assessing serum and ovarian tissue levels of ROS, TAC, hydroxyl radicals, MDA, and GSH. All transplantation groups exhibited significantly elevated levels of serum and tissue ROS, MDA, TAC, hydroxyl radical activity, and GSH compared to the control and sham groups (*p* < 0.01) (Figure 3a–f).

However, the high-dose melatonin group demonstrated significantly reduced ROS and MDA levels (Figure 3a,c,d), along with significantly increased TAC, GSH levels, and hydroxyl radical clearance ratio compared to the saline and low-dose melatonin groups (Figure 3b,e,f).

Protein expression analysis further revealed that both melatonin-treated groups exhibited a dose-dependent increase in the expression of antioxidant enzymes, including SOD and HO-1, in comparison with the saline, control, and sham-operated groups (Figure 3g–i).

### 3.4. Melatonin Protects Against Oxidative and Inflammatory Damage via the Nrf2 Pathway

To elucidate the mechanism underlying melatonin’s protective effects against oxidative and inflammatory damage, the Nrf2/antioxidant response element (ARE) signaling pathway was investigated. This pathway plays a pivotal role in cellular defense against oxidative stress, where Kelch-like ECH-associated protein 1 (Keap1) inhibits Nrf2 activity, and sMafg facilitates Nrf2 nuclear translocation, thereby promoting the transcription of antioxidant genes.

**Figure 4:**
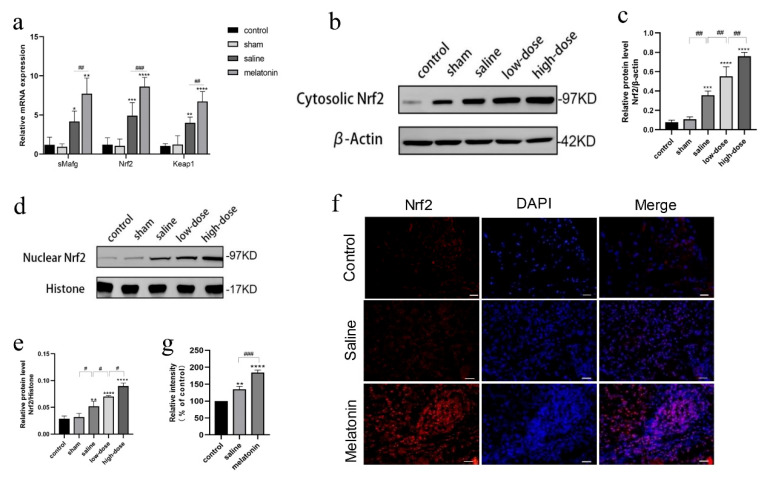
Effect of Melatonin on Keap1, sMafg, and Nrf2 mRNA and expression levels in ovarian tissue in rats receiving intact ovarian transplantation. (**a**) The expression of sMafg, Nrf2, and Keap1 mRNA in ovarian tissue in the fresh non-transplanted control, sham surgery, and saline groups. (**b**,**d**) The expression of the Nrf2 protein in the cytoplasm of ovarian tissue cells in control, sham, and melatonin-treated rats. (**c**,**e**) Western blot quantization results. (* *p* < 0.05, ** *p* < 0.01, *** *p* < 0.005, **** *p* < 0.001 vs. the control group; ^#^ *p* < 0.05, ^##^ *p* < 0.01, ^###^ *p* < 0.005 among treatment groups). (**f**) Representative immunofluorescence images showing Nrf2 expression (the red immunofluorescence signal represents Nrf2 expression; the blue immunofluorescence signal represents DAPI expression.) and (**g**) Immunofluorescence quantitative analysis (* *p* < 0.01, ** *p* < 0.001; ^###^ *p* < 0.005 vs. the control group; scale = 1000 μm). All experiments were performed in triplicate.

On postoperative day 4, significant increases in the expression of Keap1, sMafg, and Nrf2 were observed in both the saline and melatonin groups compared to the control and sham groups, with greater upregulation in the melatonin groups (Figure 4a). Moreover, Nrf2 expression levels increased significantly in a dose-dependent manner in the melatonin-treated groups relative to the other groups (Figure 4b–e). Immunofluorescence analysis further confirmed increased intensity and nuclear translocation of Nrf2 in the melatonin-treated groups (Figure 4f,g).

**Figure 5:**
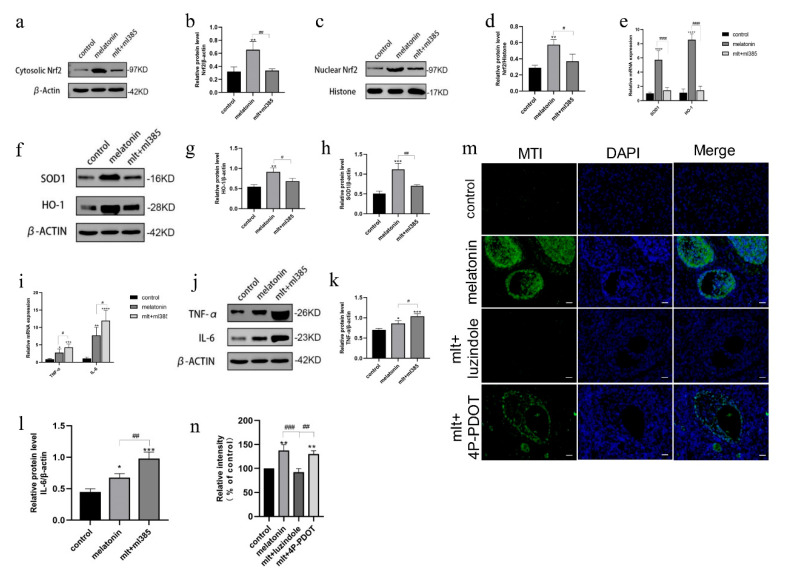
Effect of ML385 on Nrf2 levels in both cytosolic and nuclear compartments and expression levels in the ovarian tissues (**a**,**c**). Cytoplasmic Nrf2 levels in control, and upon addition of melatonin and Ml385 (**b**,**d**). Western blot quantization results. (**e**–**h**) SOD1 and HO-1 mRNA and protein expression levels in control, and upon addition of melatonin and Ml385 (**i**–**l**) TNF-α and IL-6 mRNA and protein expression in control, and upon addition of melatonin and Ml385 (* *p* < 0.005, ** *p* < 0.01, *** *p* < 0.005, **** *p* < 0.001 vs. the control group; ^#^ *p* < 0.05, ^##^ *p* < 0.01 among treatment groups). (**m**) Representative immunofluorescence images of the melatonin receptor MT1 in ovarian tissue from the fresh non-transplanted control group, melatonin, melatonin + luzindole, and melatonin + 4P-PDOT groups (the green immunofluorescence signal represents MT1 expression; the blue immunofluorescence signal represents DAPI expression). (**n**) Quantitative analysis of immunofluorescence. (** *p* < 0.01 vs. the fresh non-transplanted control group; ^##^ *p* < 0.01, ^###^ *p* < 0.005, ^####^ *p* < 0.001 between groups). Scale = 1000 μm. All experiments were performed in triplicate.

To determine whether the protective effects of melatonin were mediated via the Nrf2 pathway, rats were intraperitoneally administered a combination of melatonin and the Nrf2 inhibitor ML385. Ovarian tissues were collected over four consecutive days, and Western blot analysis confirmed a significant reduction in both cytoplasmic and nuclear Nrf2 protein levels in ML385-treated rats compared to those receiving melatonin alone (Figure 5a–d), verifying the inhibitory efficacy of ML385.

Furthermore, qRT-PCR and Western blot analysis revealed significantly increased mRNA and protein expression levels of pro-inflammatory cytokines (TNF-α and IL-6) and reduced expression of antioxidant enzymes in ML385-treated rats compared to those treated with melatonin alone (Figure 5e–l). These findings indicate that melatonin’s protective effects on transplanted ovaries were mediated through activation of the Nrf2 signaling pathway and that such effects can be suppressed by pharmacological inhibition of Nrf2.

### 3.5. Inhibition of Melatonin Receptors Impairs Ovarian Protection Mediated by the Nrf2/ARE Pathway

To determine whether melatonin activates the Nrf2 pathway via the melatonin receptor MT1, immunofluorescence detection of MT1 receptors was conducted on the fourth postoperative day. Ovarian tissue samples were collected from three groups of rats: a fresh non-transplanted control group, a melatonin-treated group (50 mg/kg), and a group treated with both melatonin and a melatonin receptor inhibitor. The results demonstrated a markedly stronger MT1 fluorescence signal (green) in the ovarian tissue of rats in the melatonin-treated group compared to the fresh non-transplanted control group. This signal was entirely abolished following the administration of luzindole, a non-selective melatonin receptor antagonist (Figure 5m). However, no reduction in MT1 fluorescence intensity was observed in the group treated with the MT2-specific antagonist 4P-PDOT. These findings suggest that melatonin may upregulate MT1 receptor expression in ovarian tissue, an effect that appears to be minimally influenced by 4P-PDOT.

**Figure 6:**
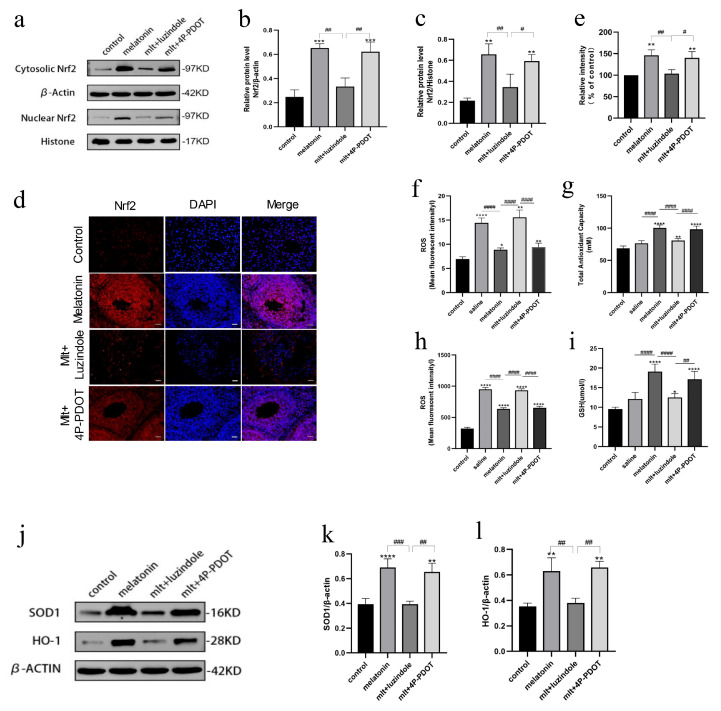
(**a**–**c**) Cytoplasmic and nuclear Nrf2 expression in the fresh non-transplanted control group, melatonin, melatonin + luzindole, and melatonin + 4P-PDOT groups. (**d**,**e**) The Nrf2 fluorescence intensity in the fresh non-transplanted control group, melatonin, melatonin + luzindole, and melatonin + 4P-PDOT groups (the red immunofluorescence signal represents Nrf2 expression; the blue immunofluorescence signal represents DAPI expression), (**e**) Quantitative analysis of immunofluorescence. (* *p* < 0.05, ** *p* < 0.01, *** *p* < 0.005, **** *p* < 0.001 vs. the fresh non-transplanted control group; ^#^ *p* < 0.05, ^##^ *p* < 0.01, ^###^ *p* < 0.005, ^####^ *p* < 0.001 between groups). Scale = 1000 μm. Levels of ROS in serum (**f**), antioxidant capacity (**g**), ROS in ovarian tissue (**h**), serum GSH levels (**i**) in the control group, melatonin, melatonin + luzindole, and melatonin + 4P-PDOT groups. SOD1 and HO-1 levels in Western blot (**j**) and their quantification (**k**,**l**) in control group, melatonin, melatonin + luzindole, and melatonin + 4P-PDOT groups. All experiments were performed in triplicate.

Subsequently, the effect of MT1 receptor inhibition on Nrf2 expression levels was evaluated. Rats received daily intraperitoneal injections of melatonin in combination with either luzindole or 4P-PDOT. Ovarian tissue samples were harvested on the fourth postoperative day for Western blot analysis. The results indicated that the expression of Nrf2 protein in both the cytoplasm and nucleus was significantly suppressed in the melatonin + luzindole group (Figure 6a–c), but remained unaffected in the melatonin + 4P-PDOT group. Considering that 4P-PDOT exhibits a 300- to 1500-fold greater affinity for the MT2 receptor [26], these findings imply that the MT1 receptor plays a predominant role in regulating Nrf2 expression in rat ovarian tissue. Immunofluorescence analysis yielded consistent results (Figure 6d,e), revealing a substantial decrease in Nrf2 fluorescence intensity in the melatonin + luzindole group compared to the melatonin-only group. In contrast, robust cytoplasmic and nuclear fluorescence signals were maintained in the melatonin + 4P-PDOT group. These results suggest that the melatonin receptor MT1 was involved in activating the Nrf2/ARE signaling pathway in cryopreserved and ovarian tissue in rats receiving intact ovarian transplantation.

### 3.6. Inhibition of Melatonin Receptors Impairs the Protective Effects of Melatonin on Oxidative Stress

To further assess the influence of receptor inhibitors on oxidative and antioxidant parameters in serum, we examined changes in serum oxidative stress markers. In the presence of the non-selective melatonin receptor antagonist luzindole, the protective effect of melatonin on serum ROS levels was reversed. ROS concentrations in the luzindole-treated group were comparable to those in the saline group and significantly higher than those observed in the melatonin-treated group (Figure 6f). No statistically significant differences in ROS levels were observed between the melatonin + 4P-PDOT group and the melatonin group (*p >* 0.05).

In the melatonin + luzindole group, serum antioxidant capacity was reduced compared to the melatonin group. It was similar to that of the saline group, whereas no significant difference was observed in the melatonin + 4P-PDOT group (Figure 6g). Similarly, ROS levels in the melatonin + luzindole group were elevated compared to the melatonin group and were comparable to those in the saline group (Figure 6h). Levels of antioxidant markers, including GSH, SOD-1, and HO-1, were reduced in the melatonin + luzindole group relative to the melatonin group, and were also comparable to levels in the saline group (Figure 6i–l). Notably, 4P-PDOT did not significantly alter these parameters.

**Figure 7:**
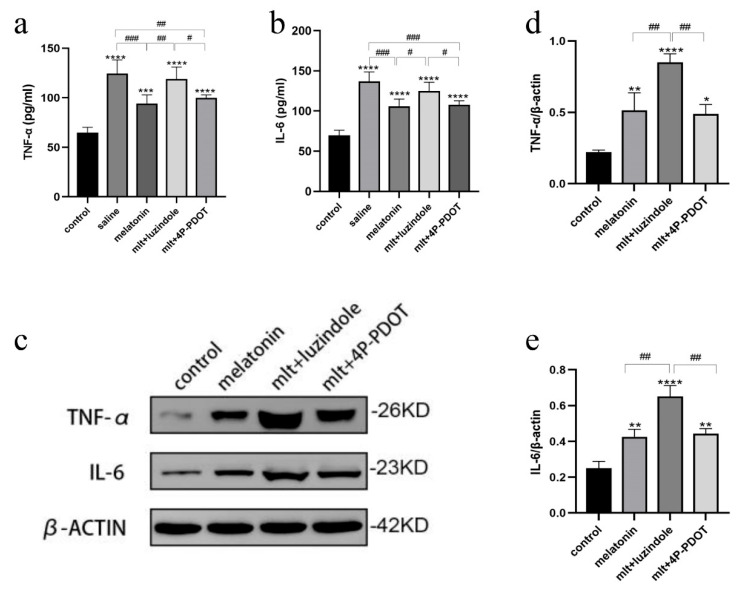
Effects of melatonin receptor antagonists on serum and ovarian tissue levels of inflammatory factors in rats receiving intact ovarian transplantation. (**a**,**b**) The levels of TNF-α and IL-6 in the serum of rats in the fresh non-transplanted control group, melatonin group, and melatonin + 4P-PDOT group. (**c**) The protein expression levels of TNF-α and IL-6 in the control group, melatonin group, and melatonin + 4P-PDOT group. (**d**,**e**) Quantitative analysis of Western blot. (compared to the control group, (* *p* < 0.05, ** *p* < 0.01, *** *p* < 0.005, **** *p* < 0.001 vs. the fresh non-transplanted control group; ^#^ *p* < 0.05, ^##^ *p* < 0.01, ^###^ *p* < 0.005 between groups). All experiments were performed in triplicate.

Changes in serum inflammatory cytokines were also assessed using ELISA. In the presence of luzindole, the protective effect observed in the melatonin-treated group was abolished, with TNF-α and IL-6 levels in the luzindole group resembling those of the saline group (Figure 7a,b). Western blot analysis confirmed these findings, revealing significantly increased protein levels of TNF-α and IL-6 in the melatonin + luzindole group compared to other groups (Figure 7c–e). Conversely, expression levels in the melatonin + 4P-PDOT group were comparable to those in the melatonin group, suggesting that the protective effects of melatonin in ovarian cryopreservation were primarily mediated through the MT1 receptor rather than MT2.

## 4. Discussion

This study demonstrates the beneficial effects of melatonin in preserving the structure and function of intact ovarian transplantation. Melatonin exerts these protective effects by modulating both inflammatory and antioxidative pathways. The administration of high-dose melatonin significantly enhances follicular reserves, maintains elevated levels of endocrine hormones such as estradiol and AMH, suppresses FSH secretion, and accelerates the resumption of the estrous cycle in recipient animals. Additionally, high-dose melatonin supplementation increases the expression of antioxidant markers, including GSH, HO-1, SOD, and total antioxidant capacity, while simultaneously reducing ROS production, MDA levels, and inflammatory cytokines such as TNF-α and IL-6 in ovarian tissue. The findings further indicate that melatonin interacts with the transmembrane receptor MT1 to initiate downstream signaling via the Nrf2/ARE pathway, thereby regulating the expression of antioxidant enzymes and inflammatory mediators to protect transplanted ovaries.

Various transcription factors, notably Nrf2, regulate the response to oxidative stress. Under physiological conditions, Nrf2 is sequestered in the cytoplasm through its interaction with the Keap1-Cul3-Rbx1 complex [27]. Upon exposure to cellular stress, Nrf2 dissociates from Keap1 and translocates to the nucleus, where it forms heterodimers with sMafg. These complexes bind to AREs in the enhancer regions of target genes, facilitating the recruitment of coactivators and initiating transcription of genes involved in cellular defense. Nrf2 is also a key regulator of antioxidative responses in the ovary, and its expression in ovarian tissues has been shown to decline with age [28,29]. In support of its antioxidative role, similar to the role of Nrf2, studies on the related factor Nrf1 knockout mice exhibit reduced numbers of primordial follicles and accelerated ovarian aging [30]. Our study employed quantitative PCR to demonstrate that melatonin significantly upregulates mRNA expression levels of sMafG, Keap1, and Nrf2 within this signaling pathway. Furthermore, a dose-dependent increase in Nrf2 protein expression was observed in response to melatonin treatment. Immunofluorescence analysis also revealed enhanced Nrf2 levels and nuclear translocation in the ovarian tissue of melatonin-treated rats. While the NRF2-mediated defense mechanism is activated under normal physiological conditions [31], its protective efficacy may be insufficient in response to severe insults such as cryopreservation and transplantation. Our findings confirm that melatonin can potentiate the activation of the Nrf2/ARE pathway, thereby enhancing tissue resilience and promoting ovarian graft survival.

To investigate whether the Nrf2/ARE signaling pathway constitutes a key mechanism through which melatonin mitigates oxidative stress and inflammatory injury in the ovarian tissue of WOCP&TP rats, the Nrf2 inhibitor ML385 was employed in combination with melatonin treatment in receptor rats. ML385 binds to the Neh1 domain of Nrf2, thereby preventing the formation of the Nrf2-sMafg complex and its binding to the ARE promoter sequence, ultimately reducing the transcriptional activity of downstream target genes [32]. In this study, administration of ML385 resulted in a significant reduction in the expression levels of Nrf2 and the downstream antioxidant enzymes SOD1 and HO-1, accompanied by a marked increase in the protein levels of the pro-inflammatory cytokines IL-6 and TNF-α. These findings suggest that the Nrf2/ARE pathway plays a critical role in enabling the ovarian tissue of rats receiving intact ovarian transplantation to counteract oxidative damage and inflammatory responses.

This study also aimed to elucidate the mechanism by which melatonin exerts its effects. Melatonin mediates its biological actions primarily through two high-affinity G protein-coupled receptors, MT1 and MT2 [33]. These receptors are widely distributed in various regions of the human central nervous system, including the suprachiasmatic nucleus (SCN), cerebellum, thalamus, and hippocampus, as well as in peripheral tissues [34]. Previous research has indicated that MT1 receptors play a more prominent role than MT2 in regulating seasonal reproduction and circadian rhythms [35]. Furthermore, MT1 receptors exhibit higher binding affinity for melatonin than MT2 receptors [26]. Accordingly, the present study focused on assessing the role of MT1 in mediating the effects of melatonin. A significant increase in MT1 receptor fluorescence signal was observed in the rats receiving intact ovarian transplantation treated with melatonin, indicating receptor activation. To precisely delineate the involvement of MT1 and MT2, the MT1/MT2 antagonists luzindole and 4P-PDOT were co-administered with melatonin in rats receiving intact ovarian transplantation. While luzindole acts as a non-selective melatonin receptor antagonist, 4P-PDOT is a selective MT2 antagonist with a 300- to 1500-fold higher affinity for the MT2 receptor [36]. Our findings showed a significant reduction in Nrf2 protein expression and nuclear translocation in rats treated with melatonin combined with luzindole, whereas melatonin combined with 4P-PDOT did not yield such effects. These results suggest that melatonin modulates Nrf2 activity primarily through the MT1 receptor.

The redox balance and inflammatory response in transplanted tissues critically influence their functionality and longevity [37]. In this study, co-administration of luzindole and melatonin led to a significant increase in ROS levels in both serum and ovarian tissue, while simultaneously reducing the total antioxidant capacity in the serum compared to the melatonin-only group. These observations further underscore the role of the MT1 receptor in mediating melatonin’s protective effects.

Previous studies have suggested that melatonin may regulate GSH levels through receptor-independent mechanisms, which are not affected by melatonin receptor antagonists [38]. However, in contrast to these findings, our study demonstrated that luzindole significantly reduced GSH levels in the ovarian tissue of receptor rats. Prior studies have also highlighted the central role of the MT1 receptor in regulating the production of inflammatory cytokines such as TNF-α, IL-1β, and IL-8 in response to melatonin [39,40,41]. In alignment with these reports, our data showed that luzindole counteracted the inhibitory effects of melatonin on the mRNA and protein expression of these cytokines. At the same time, 4P-PDOT did not exert a comparable influence. Collectively, these findings support the conclusion that melatonin confers antioxidant and anti-inflammatory protection in rats receiving intact ovarian transplantation, primarily through the MT1 receptor and the Nrf2/ARE pathway.

Although no data are available from the present study about the possible effect of melatonin on proteasomal activity, based on the literature, it is possible to hypothesize that melatonin acts as a proteasome inhibitor in various cell types. It has been shown to inhibit the chymotrypsin-like activity of the proteasome, which is essential for protein degradation in cells [42]. This inhibition is thought to occur through both direct and indirect mechanisms, including modulation of signaling pathways such as those involving calmodulin and protein kinases [42]. By inhibiting proteasomal activity, melatonin can impact the turnover of proteins regulated by the ubiquitin–proteasome system [42]. NRF2 is a transcription factor that regulates the expression of genes involved in antioxidant and cytoprotective pathways. Under normal conditions, NRF2 is ubiquitinated by the Keap1-Cul3-Rbx1 E3 ligase complex and targeted for degradation by the proteasome. Melatonin stabilizes NRF2 by inhibiting its proteasomal degradation. It leads to increased cytosolic levels of NRF2 and enhanced nuclear translocation, where NRF2 activates the transcription of ARE-driven genes [42,43,44]. The upregulation of NRF2 by melatonin results in increased expression of antioxidant enzymes such as HO-1, NQO1, SOD, and glutathione peroxidase, thereby enhancing cellular defense against oxidative stress [43,44,45]. The protective effects of melatonin against oxidative damage and inflammation are, at least in part, mediated by this stabilization and activation of NRF2 [44,45].

## 5. Clinical Practice Perspective

Ovarian tissue transplantation is an important fertility preservation technique, especially for young cancer patients at risk of premature ovarian insufficiency. However, graft survival is challenged by oxidative stress, IRI, apoptosis, and impaired revascularization [9,11,12,24,46]. Studies show melatonin promotes neoangiogenesis, which is crucial for graft integration and function [47]. Melatonin-treated grafts exhibit faster resumption of estrous cycles, higher numbers of mature follicles, and improved hormonal profiles compared to controls [24,48]. Nevertheless, there is a lack of human trials, and most evidence is derived from rodent models. There are no large-scale clinical trials in humans yet; however, a few case reports and pilot studies suggest a potential benefit [46,49,50].

## 6. Limitations

This study has several limitations. First, as an in vivo study, and the exact potential for clinical translation, particularly regarding human-equivalent doses, remains unknown. In addition, only a limited number of pathways were examined. It does not fully clarify whether melatonin’s protective effects are mediated exclusively through Nrf2 regulation or whether additional factors and signaling pathways are also involved. In the cytoplasm, Nrf2 undergoes rapid degradation following ubiquitination, thereby maintaining its protein levels at a basal level. Although previous research has suggested that melatonin may inhibit proteasomal activity [42], this aspect was not addressed in the present study. Further investigation is required to determine whether melatonin enhances cytoplasmic Nrf2 levels by inhibiting proteases. Additionally, as this study was conducted using a rodent model, further data are necessary to evaluate its translational relevance to clinical practice in humans. Finally, only the short-term outcomes were assessed. Future studies should investigate the long-term outcomes of melatonin and determine whether the same mechanisms are involved in both the acute and chronic phases after transplantation.

## 7. Conclusions

To our knowledge, this is the first study to demonstrate that melatonin protects rats receiving intact ovarian transplantation from oxidative stress and inflammatory injury induced by IRI, thereby preserving both the quality and quantity of oocytes as well as the endocrine function of the ovaries. The study also elucidated the underlying signal transduction mechanism, confirming that melatonin exerts its protective effects through activation of the MT1/Nrf2/ARE signaling pathway. These findings suggest that melatonin may represent a promising therapeutic agent for preserving ovarian graft function.

## Figures and Tables

**Table 1:** Counts of morphologically normal follicles at different stages in each group on the fourth postoperative day of rats receiving intact ovarian transplantation.

Group	Primordial Follicles	Primary Follicles	Secondary Follicles	Antral Follicles
Fresh control (*n* = 5)	103.6 ± 3.4 **	24.0 ± 2.7 **	13.4 ± 2.4 **	7.6 ± 1.1 **
Saline (*n* = 5)	56.4 ± 4.9	12.4 ± 2.1	5.6 ± 1.8	1.6 ± 1.1
low dose (*n* = 5)	64.4 ± 5.9 *	17.6 ± 2.7 *	8.4 ± 1.1 *	2.8 ± 0.8
high dose (*n* = 5)	80.8 ± 2.9 **	19.0 ± 3.5 **	10 ± 1.6 **	5.4 ± 1.1 **

Compared with the saline group, * *p* < 0.05, ** *p* < 0.01.

## Data Availability

All data generated or analyzed during this study are included in this published article.
